# CrisprPr: a hybrid-driven framework for CRISPR/Cas9 off-target prediction with analysis of prior-information updates

**DOI:** 10.1093/bib/bbag140

**Published:** 2026-03-30

**Authors:** Yingfu Wu, Yang Qi, Yiqi Chen, Dongliang Liu, Qi Liu, Xuequn Shang

**Affiliations:** School of Computer Science, Northwestern Polytechnical University, 1 Dongxiang Road, Chang'an District, Xi'an 710129, Shaanxi, China; School of Computer Science, Northwestern Polytechnical University, 1 Dongxiang Road, Chang'an District, Xi'an 710129, Shaanxi, China; School of Computer Science, Northwestern Polytechnical University, 1 Dongxiang Road, Chang'an District, Xi'an 710129, Shaanxi, China; School of Computer Science, Northwestern Polytechnical University, 1 Dongxiang Road, Chang'an District, Xi'an 710129, Shaanxi, China; Bioinformatics Department, School of Life Sciences and Technology, Tongji University, 1239 Siping Road, Yangpu District, Shanghai 200092, China; School of Computer Science, Northwestern Polytechnical University, 1 Dongxiang Road, Chang'an District, Xi'an 710129, Shaanxi, China

**Keywords:** CRISPR/Cas9 system, off-target effects prediction, prior-information, deep learning

## Abstract

CRISPR/Cas9 specificity is critically affected by off-target effects. However, the complex patterns of mismatches and their combinations at off-target sites remain difficult to capture, and existing approaches show limited capacity to identify informative features. Here, we present CrisprPr, a hybrid-driven off-target prediction framework that integrates both prior information and data-driven modeling to improve the characterization of off-target activity. CrisprPr employs a synchronous updating strategy that jointly optimizes prior-knowledge and deep-learning modules, together with multi-source integration, to deliver accurate and stable off-target predictions. Evaluations on independent test sets indicate that CrisprPr achieves competitive predictive performance and generalization compared with existing deep learning methods, with statistically significant improvements observed on several datasets. Beyond predictive performance, its analysis module examines the patterns of prior embedding-space updates to reveal distinctive target-site features supported by literature evidence. Overall, CrisprPr proposes a novel framework that demonstrates competitive predictive performance while offering new insights into the characteristics of off-target effects.

## Introduction

The clustered regularly interspaced short palindromic repeats/CRISPR-associated protein 9 (CRISPR/Cas9) system is an emerging and commonly used genome-editing technology due to its high efficiency, programmability, and operational simplicity [1]. It was initially identified as part of the adaptive immune system in bacteria and archaea [2, 3]. Currently, CRISPR/Cas9 technology is extensively applied across various disciplines such as medicine and agriculture [4–6]. For instance, CRISPR/Cas9 system can be used to construct disease models by enabling the activation or knockout of disease-related genes [7, 8], and enhance crop resilience by effectively editing genes associated with crop disease resistance [9, 10].

The CRISPR/Cas9 system comprises two primary components: the Cas9 nuclease and the single-guide RNA (sgRNA) [11, 12]. Ideally, the Cas9 nuclease is guided by the sgRNA to the target genomic site to cleave the DNA double strand, thereby achieving effective gene editing. However, off-target effects have been observed in practical applications of the CRISPR/Cas9 system [13, 14]. These effects occur when the CRISPR/Cas9 complex binds to genomic sites that are not perfectly complementary to the sgRNA, leading to unexpected genetic modifications and other potential side effects [15]. Accordingly, the clinical translation and application of the CRISPR/Cas9 system are significantly limited by the off-target effects [16]. Consequently, accurate off-target prediction, which enables the identification of sgRNAs with low off-target potential, is essential for achieving the desired genome-editing outcomes [15].

Currently, off-target prediction methods are generally categorized into two main approaches: hypothesis-driven methods and deep learning-based methods. The former methods use rules derived from limited experimental datasets to score off-target effects, such as CRISPRoff [17], MIT [14], CFD [18], and MOFF [19]. In particular, MOFF, the most recent advancement in this category, utilizes mismatch experiment results to fit parameters (e.g. single-mismatch tolerance) and applies them directly to off-target effect prediction. These methods are limited by the low complexity of their models or rules, which makes the methods more prone to overfitting. The latter methods leverage deep neural networks for modeling off-target effects. In terms of feature representation, these deep learning-based methods can be further divided into two types. The first type utilizes sparse vector encodings for sgRNA-off-target pairs, e.g. CRISPR-net [20] and CRISPR-IP [21] employ a 7-mer one-hot encoding, while CRISPR-DNT uses a 24-position encoding [22]. The second type utilizes randomly initialized learnable embedding layers to map sgRNA–off-target pairs into a continuous, low-dimensional vector space, as employed in methods such as CRISPR-OFFT [23], CRISPR-M [24], and CRISPR-DIPOFF [25]. These deep learning-based methods typically use hybrid network architectures—such as CNN-LSTM [20], CNN-Attention [23], or CNN-Attention-LSTM [21, 22, 24]—that can automatically extract sequence features related to off-target activity. Beyond the two categories outlined above, CRISOT [26] adopts a distinct strategy by characterizing RNA–DNA molecular interaction features via molecular dynamics simulations and modeling their contribution scores using XGBoost. While a variety of methods have advanced the progress of off-target prediction, a key challenge remains inherent to this task: off-target prediction inherently involves a vast combinatorial feature space (with the total number of potential base-pairing sequence combinations reaching up to $16^{20}$). This complexity leads to the limitations of existing single-source-driven methods (hypothesis-driven methods or deep learning-based methods) in capturing biologically relevant and functionally important features. In summary, existing methods remain insufficient for characterizing the complex determinants of off-target effects.

To overcome these limitations, we propose CrisprPr, a hybrid-driven off-target prediction framework. Unlike existing hypothesis-driven or deep learning-based methods, CrisprPr jointly optimizes literature-derived prior knowledge and deep learning models within a unified framework. Specifically, CrisprPr makes the following contributions: (i) We propose a hybrid-driven modeling paradigm in which literature-derived biological priors and data-driven deep learning representations jointly serve as complementary learning signals, rather than relying on existing single-source-driven approaches. (ii) We construct an ensemble off-target prediction model that integrates sub-models based on complementary mechanistic information related to off-target activity: mismatch tolerance profiles (MTP) and DNA–RNA interaction contribution scores (DRICS). (iii) We design a dedicated analysis module to characterize global similarities and differences among sequence positions and mismatch base pairs by comparing prior embedding spaces before and after model training, rather than assigning importance scores to individual features. Comprehensive cross-dataset evaluations indicate that CrisprPr achieves competitive performance compared with existing deep learning methods. Moreover, analysis of the prior embedding spaces reveals mechanistic patterns of off-target activity that align closely with experimentally observed biological phenomena.

## Methods

### Overview of CrisprPr

**Figure 1:**
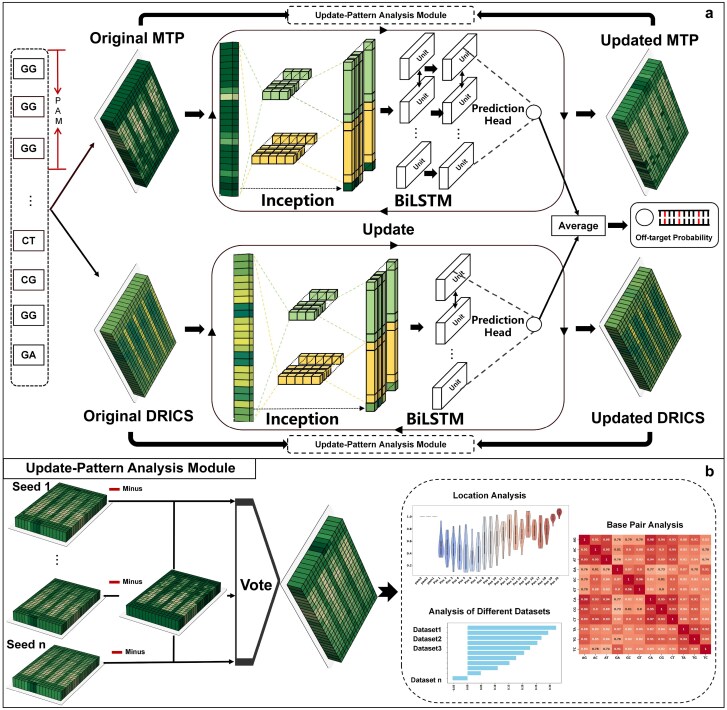
The framework of CrisprPr. (**a**) Ensemble off-target prediction module. The left side shows the original prior embedding spaces, including MTP (top) and DRICS (bottom), each separately initialized as a trainable embedding layer. The MTP and DRICS embeddings derived from the sgRNA-DNA sequence are processed by downstream deep learning modules (Inception, BiLSTM, and Prediction Head) to generate off-target probability predictions. The two branches are averaged to produce the final off-target probability. During training, the prior embeddings are updated via backpropagation (right). (**b**) Update-pattern analysis module. Embedding update patterns are analyzed from three perspectives: location analysis, base-pair analysis, and analysis across different datasets (right). In this analysis, embedding updates are computed as differences between updated and original embeddings (``Minus'') and fused across random seeds by voting (``Vote'').

The CrisprPr framework is composed of two primary modules: (1) Ensemble Off-target Prediction Module (Fig. 1a), which integrates two off-target prediction sub-models (one based on MTP prior information and the other derived from DRICS prior information) to deliver accurate and generalizable off-target predictions; (2) Update-Pattern Analysis Module (Fig. 1b), which analyzes the update pattern of the prior embedding space across sequence positions and mismatch types to reveal relevant characteristics of off-target effects.

### Data preparation

We used two public CRISPR off-target datasets, CHANGE seq [27, 28] and TTISS-CT [17, 26], for training the prediction module. The CHANGEseq dataset was selected for training due to its large sample size (2873 517 samples) and broad sgRNA coverage (110 sgRNAs), enabling extensive sequence diversity. The TTISS-CT dataset was selected as the validation set in this study for hyperparameter tuning (e.g. early stopping epochs and model architecture determination). It was generated using an experimental protocol independent of the training and test datasets, and its sgRNAs are completely nonoverlapping with those in the test sets (Supplementary Table S1), which helps avoid potential information leakage.

To assess the cross-dataset generalization ability of off-target prediction models, model evaluations were conducted on seven test datasets, including six independent test datasets and one complementary test dataset [20, 22]. The six independent test datasets differ from the CHANGEseq dataset in terms of cell types and off-target detection technologies. Based on the data composition, these datasets can be broadly categorized into three evaluation settings: (i) the HEK293T and K562 datasets each correspond to a single cell type and are profiled using multiple detection technologies [29]; (ii) the Guide-CT and II/6 datasets contain data from multiple cell types measured using a single detection technology [26, 30, 31]; (iii) the CRISPOR and PDH datasets contain data from multiple cell types and multiple detection technologies [32, 33]. For the six independent test datasets, sgRNAs duplicated or exhibiting sequence similarity $\ge 90\%$ to those in the CHANGEseq training set were excluded to ensure independence between training and testing data (Supplementary Table S1). In addition, sgRNAs reported in source studies used for constructing biological prior information were filtered out to further reduce the risk of information leakage. The GUIDEseq dataset was included as a complementary test dataset, in which sgRNA sequences are shared with the CHANGEseq training data while experimental conditions differ [34]. Detailed information for each dataset is provided in Table 1.

**Table 1:** Summary of datasets.

	Dataset	Number of sgRNA	Number of positive	Number of negative	Total number of samples	Technique	Cell type	reference
Train	CHANGEseq	110	67 369	2806 148	2873 517	CHANGE-seq	human primary T	[27, 28]
Val	TTISS-CT	59	866	668 782	669 648	TTISS-seq	U2OS	[17, 26]
Test	HEK293T	16	489	128 146	128 635	GUIDE-seq Digenome-seq BLESS HTGTS IDLV	HEK293T	[29]
	K562	11	112	17 673	17 785	GUIDE-seq Digenome-seq BLESS HTGTS IDLV	K562	[29]
	Guide-CT	30	366	320 178	320 544	GUIDE-seq	U2OS HEK293	[26, 30]
	CRISPOR	26	657	367 819	368 476	GUIDE-seq CIRCLE-seq Digenome-seq HTGTS	U2OS HEK293T K562 HAP1	[32]
	II/6	22	56	383 385	383 441	GUIDE-Seq	U2OS HEK293T K562 Kbm7 HL60 HCT116	[31]
	PDH	7	328	245 091	245 419	Digenome-Seq PCR HTGTS	HAP1 K562	[33]
	GUIDEseq	57	1583	1455 289	1456 872	GUIDE-Seq	U2OS	[34]

### Construction of embedding spaces based on prior information

Given the complexity of off-target features, randomly initialized embedding spaces have limited capacity to capture key determinants of off-target activity. In this study, we derived reported information related to off-target effects from existing studies to construct prior-informed embedding spaces, thereby enhancing feature learning. Firstly, the initial embedding space of the MTP was constructed using the tolerance prior information for different mismatch types at various positions in real off-target sites, which was reported in the study by Fu *et al*. [19]. Let $L$ denote the length of both the sgRNA and the off-target sequence. The pairing type at each position is drawn from a finite set $\beta$ of size $|\beta |$, including matching base pairs (AA, CC, GG, TT) and mismatch base pairs (AC, AG, AT, CA, CG, CT, GA, GC, GT, TA, TC, TG). The initial embedding space can therefore be represented as a matrix $M^{\textrm{init}} \in R^{L\times \beta },\ L=24,\ |\beta |=16$, where $M_{i,j}^{\textrm{init}}$ represents the tolerance score of the $j$th base-pair type at the $i$th position.

Since the tolerance information for all base-pair types in the PAM region (essential for Cas protein binding and cleavage initiation; Fig. 1a) and for matching types in the other region is absent in the literature, we set the initial values for these missing entries to 1, thereby completing the initial embedding space of MTP. The initial embedding space of MTP, defined by the element-wise rules below, is represented as


1 \begin{eqnarray*}& M_{i,j}^{\textrm{init}}= \begin{cases} 1, &i \in \textrm{pam and}\ j \in \beta \\ 1, &i \not\in \textrm{pam and}\ j \in \{AA, TT, \\ &CC, GG\} \\ \textrm{known tolerance value}, &\textrm{otherwise}. \end{cases}\end{eqnarray*}

Secondly, the initial embedding space of the DRICS was constructed using prior information on the contribution scores of different types of RNA–DNA base pair interaction characteristics at each sequence position, as reported by Chen *et al*. [26]. Similar to $M^{\textrm{init}}$, the initial embedding space of DRICS can be represented as $D^{\textrm{init}} \in R^{L\times \beta }$. Similarly, contribution scores for all possible base-pair types in the PAM region (Fig. 1a) were set to 0, owing to the absence of corresponding information in the literature. The resulting initial embedding space of DRICS is represented as


2 \begin{eqnarray*}& D_{i,j}^{\textrm{init}}= \begin{cases} 0, &i \in \textrm{pam and}\ j \in \beta \\ \textrm{known contribution score}, &\textrm{otherwise}. \end{cases}\end{eqnarray*}

In the study by Chen *et al*., the contribution scores range from −5.5 to 0.6. Specifically, scores for matching types are predominantly concentrated in the positive interval (0, 0.6), whereas those for mismatch types are mainly distributed in the negative interval (−5.5, 0). To preserve the distinction between matched and mismatched pairs (positive versus negative) and the relative distances within matched and mismatched base pairs, we adopt a piecewise normalization strategy. Accordingly, the overall DRICS values are normalized to address the range differences between the positive and negative intervals. Specifically, the values in the positive and negative intervals were normalized to [0, 1] and [−1, 0], respectively:


3 \begin{eqnarray*}& D_{i,j}^{\textrm{init, norm}}= \begin{cases} D_{i,j}^{\textrm{init}} / v_{max}, &if\, D_{i,j}^{\textrm{init}} \ge 0 \\ D_{i,j}^{\textrm{init}} / |v_{min}|, &if\, D_{i,j}^{\textrm{init}} < 0, \end{cases}\end{eqnarray*}

where $v_{max}$ and $v_{min}$ are the maximum and minimum contribution scores, respectively.

### Ensemble deep learning model for off-target prediction

To overcome the limitations of a single-source driving strategy in capturing off-target features, we adopted a hybrid-driven approach that combines deep learning with prior embedding space. First, the deep learning prediction models based on the MTP and DRICS prior embedding spaces, which served as sub-models of the ensemble model, were constructed as follows.

#### Off-target prediction sub model based on MTP

Based on MTP prior information, we constructed a deep learning-based off-target prediction sub-model, termed M-model. The first layer of M-model is a trainable MTP embedding space, in which the sgRNA–DNA sequence is encoded by indexing with position (row) and pairing type (column). The remaining architecture of the M-model was adapted from the CRISPR-net framework [20], which has demonstrated strong performance in multiple prior studies [19, 21, 28]. The CRISPR-net architecture integrates an Inception module with a bidirectional LSTM (BiLSTM) to capture both local and global sequence features.

The Inception module was used for local feature extraction, consisting of three 1D convolution branches with kernel sizes of 3, and 5 (20 kernels each), together with a residual branch for identity mapping. To capture long-range dependencies, the BiLSTM module comprises two bidirectional LSTM layers, each with a hidden size of 25. These structural parameters were optimized based on the validation AUPRC results (Supplementary Figure S1a, b). After the Inception and BiLSTM modules, the flattened features are passed through three fully connected layers, comprising 80, 20, and 1 neurons, respectively, to output the predicted off-target probability. The MTP embedding space is trainable and is updated synchronously with the downstream Inception and BiLSTM modules during backpropagation. Detailed model architecture and training details are provided in the Supplementary Methods.

#### Off-target prediction sub model based on DRICS

Similarly, an off-target effect prediction sub-model, D-model, was constructed based on DRICS prior information, in which Inception and BiLSTM modules were also used for feature extraction. In D-model, the Inception module consists of 1D convolutions with kernel sizes 3, and 5 (20 kernels each), along with a residual branch for identity mapping. The BiLSTM module comprises one layer with a hidden size of 20. These structural parameters were optimized based on validation results following the same procedure as applied to the M-model (Supplementary Figure S1c, d). In addition, the DRICS embedding space is implemented as the first layer of D-model and is jointly optimized with Inception and BiLSTM modules during training. Model architecture and training details are in the Supplementary Methods.

Finally, because MTP and DRICS prior information originate from different sources and represent distinct information, we employed an average fusion strategy to combine the outputs of M-model and D-model, thus constructing an ensemble model for off-target prediction.

### Update-pattern analysis module

To further analyze the underlying patterns of off-target effects, we constructed a systematic analysis module based on the prior embedding space before and after the update. First, considering the differences in neural network initialization across random initialization seeds, a voting strategy was used to fuse the variations in embedding space updates based on multiple random initialization seeds. The variation in the embedding space updates based on each random seed is calculated as follows:


4 \begin{eqnarray*}& \begin{split} \Delta M_{s, i, j} &= M_{s, i, j}^{\textrm{update}} - M_{i, j}^{\textrm{init}},\\[-2pt] i &= 1,\dots,L,\quad j = 1,\dots,|\beta|,\quad s = 1,\dots,n, \end{split}\end{eqnarray*}

where $M_{i,j}^{\textrm{init}}$ denotes the initial embedding value of base-pair type $j$ at position $i$, and $M_{s,i,j}^{\textrm{update}}$ denotes the updated value obtained from the model initialized with random seed $s$. Voting was performed based on the variation trend $t_{s, i, j}$ of each point in the embedding spaces updated by models initialized with different initialization seeds. If the variation trend $t_{s, i, j}$ corresponds to an increase (i.e. $\Delta M_{s, i, j}$ is greater than 0), then the value is assigned as 1; conversely, it is assigned as −1. If the variation trend is consistent across more than half of the initialization seeds, we define this trend as the primary direction of change at that position and denote it by $T_{i, j}$:


5 \begin{eqnarray*}& T_{i, j}= \begin{cases} 1, &\left(\sum_{s=1}^{n}t_{s, i, j}\right)\ge 1 \\ -1, &\left(\sum_{s=1}^{n}t_{s, i, j}\right)\le -1 \end{cases}\end{eqnarray*}

After determining the primary direction of change at each position, the corresponding change values from all random initialization seeds whose directions match the primary direction were aggregated. Specifically, the fused change value $\Delta M_{i, j}^{\textrm{Fusion}}$ is computed as the mean of these selected change values.


6 \begin{eqnarray*} & S_{i,j}=\{s\in{1,...,n}\ |\ t_{s, i, j}=T_{i, j}\} \end{eqnarray*}

7 \begin{eqnarray*} & \Delta M_{i, j}^{\textrm{Fusion}}=\frac{1}{|S_{i,j}|}\sum\limits_{s\in S_{i,j}}\Delta M_{s, i, j}, \end{eqnarray*}

where $S_{i,j}$ denotes the set of random initialization seeds whose change directions are consistent with $T_{i, j}$. The final fused embedding space is given by


8 \begin{eqnarray*}& M^{\textrm{Fusion}}=M^{\textrm{init}} + \Delta M^{\textrm{Fusion}}.\end{eqnarray*}

We used violin plots to compare the distributions of embedding values associated with mismatch types at each position in the original and updated embedding spaces($M^{\textrm{init}}$ and $M^{\textrm{Fusion}}$). To examine the differences among mismatch base-pair types, we performed a similarity analysis on their embedding distributions. Considering the inherent differences between similarity measures, the Euclidean distance and Pearson correlation coefficient were weighted and fused to comprehensively evaluate the similarity between mismatch base-pairs:


9 \begin{eqnarray*}& \Phi_{p_{1},p_{2}}=(1-\alpha)\cdot P_{p_{1},p_{2}} + \alpha\cdot (1-Norm(E_{p_{1},p_{2}})),\end{eqnarray*}

where $P_{p_{1},p_{2}}$ and $E_{p_{1},p_{2}}$ denote the Pearson correlation coefficient and the Euclidean distance of the distributions of mismatch types $p_{1}$ and $p_{2}$, respectively. $Norm()$ denotes the normalized operation. $\alpha$ is the weight coefficient and was set to 0.5.

### Evaluation criteria

In real experiments, off-target effects are usually verified for a single sgRNA to distinguish real off-target sites from potential off-target sites. Therefore, we adopted a single sgRNA-based evaluation strategy to assess the performance of the proposed model in this study. Specifically, for each sgRNA with true off-target sites in the test set, we computed the Area Under the Precision–Recall Curve (AUPRC) score, denoted as $Sg{\_}AUPRC$, and the Area Under the Receiver Operating Characteristic Curve (AUROC) score, denoted as $Sg{\_}AUROC$. Then the $Sg{\_}AUROC$ and $Sg{\_}AUPRC$ values were averaged across all sgRNAs to evaluate the overall performance of the method on the entire test dataset:


10 \begin{eqnarray*} & AUROC=\frac{1}{N}\sum\limits_{r=1}^{N}Sg{\_}AUROC_{r} \end{eqnarray*}

11 \begin{eqnarray*} & AUPRC=\frac{1}{N}\sum\limits_{r=1}^{N}Sg{\_}AUPRC_{r}, \end{eqnarray*}

where $r$ denotes the $r$th sgRNA, and $N$ denotes the number of sgRNAs with true off-target sites in the test set. Given the imbalance of the test datasets and the critical importance of identifying true off-target sites, AUPRC was used as the primary evaluation metric [21, 25, 35], with AUROC as a complementary measure. Precision, recall, F1-score, and Matthews correlation coefficient (MCC) were additionally reported as supporting evaluation metrics. These metrics were computed at the sgRNA level and aggregated within each dataset, as with AUPRC and AUROC. Statistical significance of performance differences between methods was assessed with a one-sided Wilcoxon signed-rank test across multiple random initializations.

## Results

### Comparison with state-of-the-art methods on test sets

To comprehensively evaluate the effectiveness and the performance advantages of our proposed CrisprPr prediction module, we employed single sgRNA-based evaluation on seven test sets (see Methods section for details). We further compared CrisprPr with state-of-the-art off-target prediction methods including MOFF [19], CRISOT [26], CRISPR-OFFT [23], CRISPR-net [20], CRISPR-IP [21], CRISPR-DNT [22], CRISPR-M [24], and CRISPR-DIPOFF [25]. All deep learning methods (i.e. CRISPR-OFFT, CRISPR-net, CRISPR-IP, CRISPR-DNT, CRISPR-M, and CRISPR-DIPOFF) were trained on the CHANGEseq dataset and validated on the TTISS-CT dataset for early stopping. Considering the influence of random initialization seeds on deep learning models, we conducted five independent experiments with different random initializations, and the evaluation results obtained from these experiments were used for performance comparison and stability analysis. Moreover, the CRISOT and MOFF methods were evaluated using original parameters reported in their study, which were derived from specialized fitting procedures. The comparative performance of the evaluated methods across datasets is summarized below.

**Figure 2:**
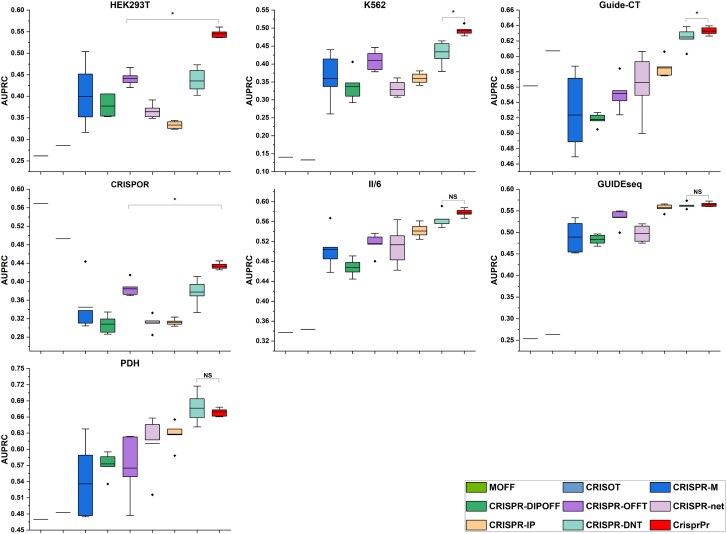
Comparison of CrisprPr with state-of-the-art methods on seven test datasets in terms of AUPRC. The compared methods are ordered as follows: MOFF, CRISOT, CRISPR-M, CRISPR-DIPOFF, CRISPR-OFFT, CRISPR-net, CRISPR-IP, CRISPR-DNT, and CrisprPr. The central line represents the mean value across five independent experiments, while the bottom and top of the vertical line denote the minimum and maximum values, respectively. Significance: $*, P <.05$; $NS, P \ge .05$ (Wilcoxon signed-rank test).

Among the compared methods, traditional (non-deep learning) approaches such as MOFF and CRISOT exhibit substantial performance variation across datasets, reflecting limited cross-dataset stability (Supplementary Figure S2). Both methods achieve strong performance on only a small number of datasets, while their performance varies considerably on others. Overall, deep learning-based methods exhibit relatively more consistent performance across datasets compared with traditional approaches. Therefore, comparisons are focused on deep learning-based methods, with AUPRC used as the primary evaluation metric. CrisprPr achieves the best or statistically comparable AUPRC on all seven datasets, with statistically significant improvements on four datasets, one case approaching statistical significance, and comparable performance on two datasets (Fig. 2). Specifically, CrisprPr significantly outperforms competing deep learning methods on HEK293T (0.5450 versus 0.4359, $P$ =.031), K562 (0.4931 versus 0.4341, $P$ =.031), Guide-CT (0.6328 versus 0.6252, $P$ =.031), and CRISPOR (0.4334 versus 0.3846, $P$ =.031). On II/6, CrisprPr shows a consistent numerical improvement in AUPRC (0.5778 versus 0.5649, $P$ =.06), though the difference is not statistically significant. Comparable AUPRC performance is observed on GUIDEseq (0.5641 versus 0.5626, $P$ =.406) and PDH (0.6693 versus 0.6765, $P$ =.688). For both F1-score and MCC, CrisprPr achieves statistically significant improvements over the strongest competing deep learning methods on five out of seven datasets, while remaining comparable on the others (Supplementary Figures S3 and S4). Substantial differences are observed among methods with respect to precision and recall, indicating heterogeneous prediction behaviors (Supplementary Table S2). AUROC values are consistently high ( $\ge 0.9$) for all methods, with only small variations observed across models. Across datasets, CrisprPr achieves AUROC values within a narrow range of the top-performing method (Supplementary Figure S5).

In addition, model stability was evaluated by computing the variance of AUPRC across five random initializations. Across datasets, CrisprPr achieves lower AUPRC variance than other deep learning methods on five out of seven datasets (Supplementary Tables S3). Taken together, these results indicate that CrisprPr achieves competitive predictive performance and exhibits improved stability across multiple independent datasets compared with existing deep learning methods.

### Ablation analysis of CrisprPr

To evaluate the effectiveness of the synchronous updating strategy in the CrisprPr framework, we constructed Noupdate-M-model and Noupdate-D-model, derived from the M-model and D-model with the prior embedding space update disabled. The architecture and training process of the two control models were aligned with those of the M-model and D-model. The performance of models was also compared across seven test datasets. Furthermore, to assess the contribution of the integration strategy, we compared the results of the M-model, D-model, and the CrisprPr ensemble model.

**Figure 3:**
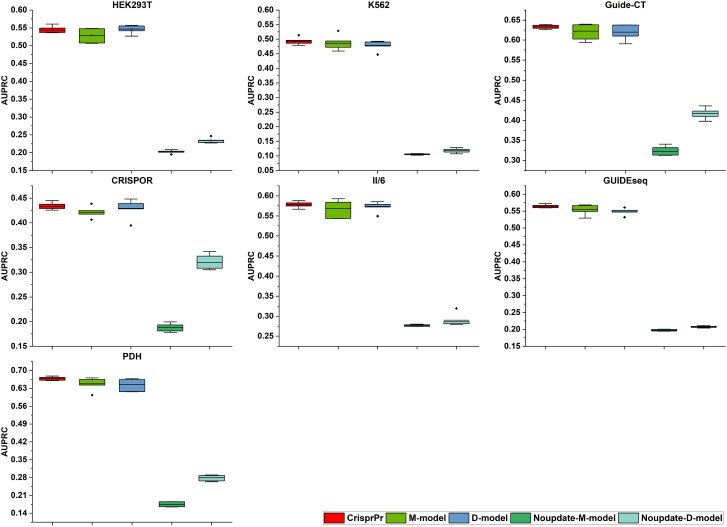
Comparison of CrisprPr with its ablation models on seven test datasets in terms of AUPRC. The compared methods are ordered as follows: CrisprPr, M-model, D-model, Noupdate-M-model, and Noupdate-D-model. The central line represents the mean value across five independent experiments, while the bottom and top of the vertical line denote the minimum and maximum values, respectively.

The results of M-model versus Noupdate-M-model and D-model versus Noupdate-D-model demonstrate that, across all test datasets, the predictive performance of the models without the synchronous updating mechanism is significantly lower than that of their corresponding counterparts (Fig. 3 and Supplementary Figure S6). This observation indicates that the initial prior distribution deviates from the actual off-target effects. However, the synchronous updating strategy implemented in CrisprPr effectively guides the prior distribution to approximate the true off-target distribution, thereby enhancing the model’s feature representation capacity and generalization performance. Furthermore, the comparison between CrisprPr with M-model and D-model demonstrates that CrisprPr generally outperforms the two sub-models across the seven test datasets (Fig. 3 and Supplementary Figure S6), supporting the effectiveness of the mean integration strategy in improving off-target prediction performance.

To evaluate the effectiveness of the Inception and BiLSTM modules, we constructed the following control models: (i) M-model and D-model ablation variants without the Inception module (denoted as M-w/o-Inception and D-w/o-Inception); (ii) M-model and D-model ablation variants without the BiLSTM module (denoted as M-w/o-BiLSTM and D-w/o-BiLSTM). Similarly, we compared each ablation variant with its corresponding original model across seven test datasets.

**Figure 4:**
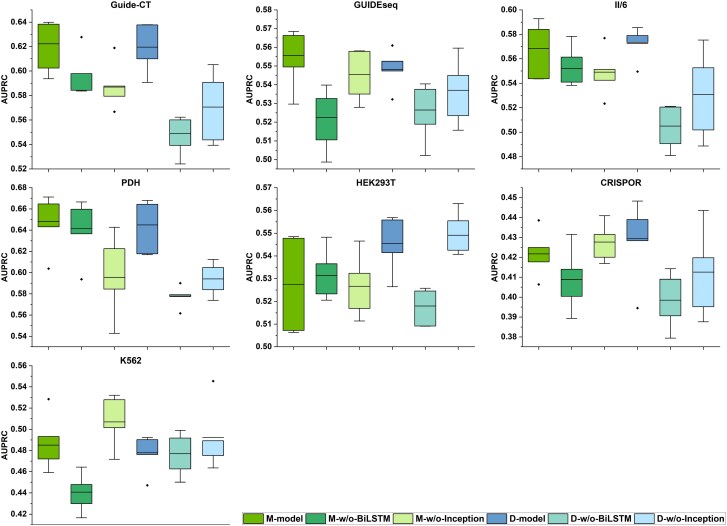
Comparison of M-model and D-model with their module-ablated variants on seven test datasets in terms of AUPRC. The compared methods are ordered as follows: M-model, M-w/o-BiLSTM, M-w/o-Inception, D-model, D-w/o-BiLSTM, and D-w/o-Inception. The central line represents the mean value across five independent experiments, while the bottom and top of the vertical line denote the minimum and maximum values, respectively.

The comparison results are shown in Fig. 4 and Supplementary Figure S7. Based on the AUPRC evaluation, both M-model and D-model exhibit better overall performance than their ablated variants (w/o BiLSTM and w/o Inception) across the test datasets. Therefore, the complete architectures integrating both Inception and BiLSTM modules show superior ability in identifying true off-target sites, achieving the best overall predictive performance. Furthermore, the performance differences shown in Fig. 4 indicate that removing the BiLSTM module leads to a larger decrease in AUPRC than removing the Inception module. This observation suggests that long-range dependencies also play a role in off-target prediction, indicating that both local and distal mismatch combinations contribute to the occurrence of off-target effects [19]. Together, these findings indicate that architectures integrating both Inception and BiLSTM modules achieve competitive or better performance across most datasets, consistent with the complementary roles of local and long-range feature modeling.

### Analysis of update patterns in the prior embedding space

Previous experimental results demonstrate that the synchronous updating strategy can effectively optimize the distribution of the prior embedding space. Therefore, to further reveal the significant update patterns of the prior embedding space, we conducted a systematic analysis between the original and updated distributions (see Methods section for details, Fig. 1b). First, we analyzed update patterns across all mismatch types at each position in the MTP embedding space by comparing the original and updated embeddings.

**Figure 5:**
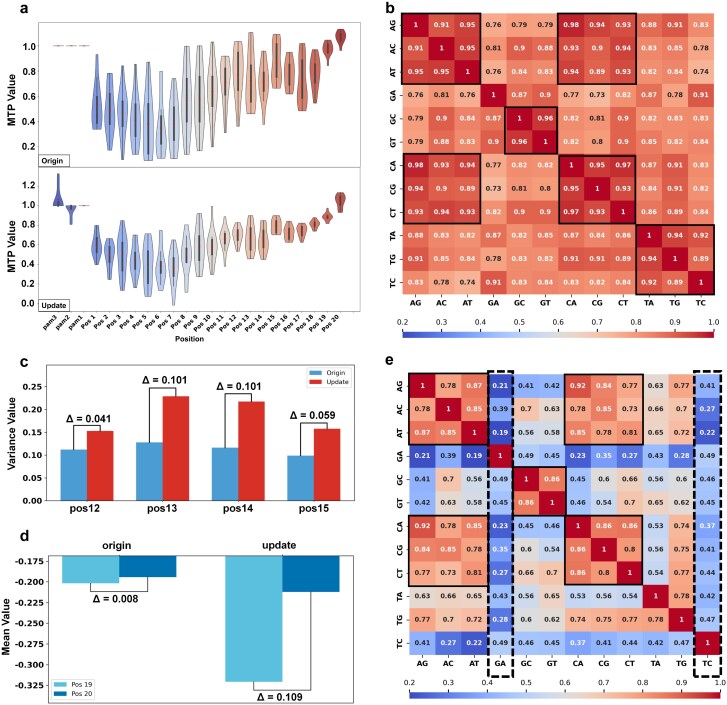
Visualization of update patterns in the prior embedding space. (**a**) Comparison of original and updated distributions of all mismatches at different positions in the MTP embedding space. (**b**) Heatmap of mismatch base-pair similarity in the PAM-distal non-seed region of the updated MTP embedding space. (**c**) Comparison of variance in mismatch distributions at positions 12–15 between the original and updated DRICS embedding spaces. $\Delta$ denotes the absolute difference between adjacent bars (|Update—Origin|). (**d)** Comparison of mean mismatch distributions at positions 19 and 20 between the original and updated DRICS embedding spaces. $\Delta$ denotes the absolute change in the mean value between corresponding groups (|Pos20–Pos19|). (**e**) Heatmap of pairwise similarity between base-pair types derived from the updated DRICS embedding space. In **(b)** and **(e)**, similarity was assessed using a weighted combination of Euclidean distance and Pearson correlation coefficient, and base-pair groups were defined as (AG, AC, AT) = rA, (GA, GC, GT) = rG, (CA, CG, CT) = rC, and (TA, TG, TC) = rT; TC = rU–dG and GA = rG–dT.

Comparative analysis shows that the updated MTP embedding distributions exhibit the following characteristic patterns of variation (Fig. 5a): (i) compared with the original distributions, the updated distributions in the positions 11–20 region are overall more compact, exhibiting a distinct divergence from the positions 1–10 region. This observation is in agreement with prior findings that define positions 1–10 as the PAM-proximal seed region, in contrast to positions 11–20 (the PAM-distal non-seed region) [36]. Further, it supports established conclusions regarding the differential mismatch sensitivity between PAM-proximal and PAM-distal sites [14, 37]. (ii) The updating process substantially reduced the variance of the distributions at positions 1 and 2, indicating a mismatch tolerance pattern distinct from that of other sites within the PAM-proximal seed region. This observation is consistent with conclusions from previous studies, which have reported a distinct mismatch tolerance pattern at these positions. Specifically, base pairs at positions 1 and 2 lack direct Cas9 contacts and thus experience fewer steric constraints than other seed positions [38, 39]. (iii) In the updated embedding space, the distributions at positions 13 and 14 exhibit increased dispersion compared with positions 12 and 15–20, indicating a distinct pattern. This observation aligns with prior reports that mismatches at positions 13–14 can modulate Cas9 cleavage activity [38]. Notably, structural studies indicate that the REC3 domain of Cas9 does not make direct contacts with nucleotides in this region, which may underlie a distinct mechanism of mismatch sensing or tolerance [40]. Moreover, although positions 19 and 20 have commonly been discussed together in previous studies [40, 41], our analysis revealed an increased divergence between the distributions at positions 19 and 20 after the updating process. This divergence may be associated with R-loop formation, where the free-energy landscape indicates that position 19 resides in a transition regime, whereas position 20 corresponds to a stable energy minimum [42].

To analyze the differences among mismatch base-pair types, we assessed pairwise similarities between mismatch base-pair type distributions in the PAM-proximal seed and PAM-distal non-seed regions of the original and updated MTP embedding spaces (Fig. 5b, Supplementary Figures S8 and S9). The results indicate that in the PAM-distal non-seed region, although the overall similarity increases, the intra-group similarities of rA, rG, rC, and rT become more pronounced relative to other similarities (Fig. 5b and Supplementary Figure S8), which suggests that mismatch types sharing the same gRNA base exhibit stronger distributional similarity. This finding is in partial agreement with the guide-intrinsic mismatch tolerance effect previously reported in the literature [19, 43]. In addition, a notable similarity was observed between rA and rC, suggesting that mismatches involving these two gRNA bases may share a common underlying mechanism in the off-target effects.

We performed the same analysis on the DRICS embedding space. In the DRICS space, the most evident variation in mismatch distributions across positions is characterized by slightly higher dispersion at positions 13 and 14 compared with neighboring sites (Fig. 5c), and increased divergence between positions 19 and 20 (Fig. 5d), consistent with the update patterns in the MTP space. The heatmap of base-pair similarity in the DRICS space shows that although the overall similarity decreased, both the intra-group similarity of rA and rC and the inter-group similarity between rA and rC emerge as more distinct patterns (Fig. 5e and Supplementary Figure S10). In contrast to MTP, the updated DRICS embedding space shows that TC (rU-dG) and GA (rG-dT) exhibit universally low similarity to all other base-pair types. This phenomenon is likely attributable to the ability of rU-dG and rG-dT to form distinctive wobble base-pair structures [39, 44], which influence off-target effects in a distinctive manner. Such distinctive similarity patterns can ultimately be explained by the fact that DRICS incorporates the physical structural characteristics of RNA–DNA base pairs into its prior information, information not encompassed in MTP. These findings demonstrate that although different types of prior information stably enhance the predictive performance for off-target effects, the update patterns of the embedding spaces reveal both shared and distinct features.

### Differential analysis of different datasets

To analyze the differences in prior information update patterns across datasets, we trained models on nine datasets and derived the embedding space update distributions for each dataset. Pearson correlation coefficients were used to quantify pairwise similarities of update distributions across datasets. For each dataset, the mean similarity with all others was computed as an overall consistency measure, and datasets were ranked accordingly (Supplementary Figure S11, Supplementary Tables S4 and S5). Although CHANGEseq is the largest dataset, its update similarity differs across embedding spaces: highest in MTP but considerably lower in DRICS. In contrast, the Guide-CT, PDH, HEK293T, and GUIDEseq datasets exhibit consistently high concordance in their similarity rankings of update distributions across both the MTP and DRICS embedding spaces (Supplementary Figure S11). This observation may suggest that Guide-CT, PDH, HEK293T, and GUIDEseq datasets contain more comprehensive information, which facilitates more consistent updates across different sources of prior knowledge.

### Discussion

CrisprPr introduces a hybrid-driven framework that integrates literature-derived prior knowledge with deep learning for CRISPR/Cas9 off-target prediction. By synchronously updating prior embedding spaces and neural network parameters, CrisprPr allows prior assumptions to be refined by empirical data rather than treated as fixed constraints, thereby establishing a hybrid modeling paradigm that bridges hypothesis-driven and purely data-driven approaches.

Results of multiple methods on the GUIDEseq dataset indicate that, even with identical sgRNA sequences, differences exist across off-target detection technologies, highlighting the importance of model generalization (Fig. 2). Against this background, CrisprPr shows competitive performance compared with existing deep learning methods across multiple cross-dataset evaluations conducted on independent test sets spanning diverse cell types and off-target detection technologies. This stability may be attributed to the incorporation of literature-derived prior knowledge, which biases the model toward sequence patterns associated with off-target activity and reduces reliance on dataset-specific patterns. It is worth noting that, for HEK293T, CRISPOR, and K562, the M-w/o-Inception variant exhibited AUPRC values ranging from comparable with higher relative to the full M-model. Our dataset-level analysis reveals a negative trend between the mean distance of adjacent mismatches and the performance change induced by the Inception module (Supplementary Figure S12). This observation suggests that mismatch distance distributions in real off-target sites may influence the effectiveness of local feature extraction and sequential context modeling.

The analysis module further demonstrates the effectiveness of updating prior embeddings during training. The observed changes in the prior embedding space are structured rather than arbitrary. These changes remain consistent with established off-target patterns and highlight position- and mismatch-specific structures after optimization.

To assess the sensitivity of CrisprPr to prior encoding initialization, binary-value (0/1) and mean-value initialization were compared. No statistically significant differences in predictive performance were observed across datasets, indicating that the main conclusions of this study are robust to reasonable variations in prior initialization. Nevertheless, marginal differences in average performance were observed (Supplementary Figure S13), with mean-value initialization showing slightly higher average performance for the DRICS-based sub-model, while no consistent difference was observed for the MTP-based sub-model. Beyond initialization choices, training data selection has also been shown to influence sub-model optimization. Notably, a larger dataset does not invariably represent the optimal choice for training prior-informed deep learning sub-models, highlighting the role of dataset composition. Taken together, these observations motivate future studies to clarify how dataset composition, prior information initialization, and the update strategies of deep learning modules interact to influence predictive performance.

## Conclusion

In this study, we proposed a novel off-target prediction framework, CrisprPr. CrisprPr introduces a new paradigm by combining literature-reported prior knowledge with deep learning models to enable prediction and analysis of off-target effects. Rather than using prior information as static features, CrisprPr proposes a synchronous updating strategy that enables the joint optimization of both the prior embedding space and the deep learning modules. To fully leverage the multi-source literature prior knowledge, CrisprPr integrates the corresponding prior information-deep learning sub-models to obtain the final prediction results. In this work, cross-dataset and ablation experiments provide supporting evidence for the competitive predictive performance of CrisprPr in off-target prediction and the effectiveness of its architecture. Through comparative analysis of the prior embedding space before and after updating, the analysis module of CrisprPr reveals characteristic patterns related to actual off-target effects, such as distinctive positions and mismatch similarity-difference. Overall, this work contributes a novel paradigm for model construction in CRISPR/Cas9 off-target prediction, while the analysis module offers additional insights into the underlying mechanisms of off-target effects through the characterization of updating patterns.

**Key Points**

- We present CrisprPr, a hybrid-driven framework that couples biological knowledge with data-driven deep learning to improve CRISPR/Cas9 off-target prediction.
- By leveraging complementary mechanistic information on mismatch tolerance and DNA–RNA interactions, CrisprPr achieves competitive predictive performance across multiple datasets.
- Beyond prediction accuracy, CrisprPr provides mechanistic insights into off-target activity through analysis of learned embedding spaces informed by biological knowledge.

## Supplementary Material

Supplementary_Information_bbag140

## Data Availability

All data used in this study are publicly available and are referenced in the manuscript. All code and data for CrisprPr are available at https://github.com/mochew/CrisprPr.
