# Supplementary material for: CrisprPr: a hybrid-driven framework for CRISPR/Cas9 off-target prediction with analysis of prior-information updates
Source: Brief Bioinform. 2026 Mar 30;27(2):bbag140. doi: 10.1093/bib/bbag140 (PMC13034848; doi:10.1093/bib/bbag140)
Supplement: Supplementary_Information_bbag140 [file supplementary_information_bbag140.pdf]

Supplementary information

CrisprPr: A Hybrid-Driven Framework for CRISPR/Cas9 Off-Target Prediction with Analysis of Prior-Information Updates

Yingfu Wu[1], Yang Qi[1], Yiqi Chen[1], Dongliang Liu[1], Qi Liu[2,*], Xuequn Shang[1,*]

[1*]School of Computer Science, Northwestern Polytechnical University, Xi'an, 710072, Shaanxi, China.

[2] School of Life Sciences and Technology, Tongji University, Shanghai, 200092, China.

*Corresponding author(s). E-mail(s): qiliu@tongji.edu.cn; shang@nwpu.edu.cn;

Table of Contents

Supplementary Methods 3
Supplementary Figure S1. Hyperparameter optimization of the M-model (a,b) and D-model (c,d) architectures 6
Supplementary Figure S2. Performance gap of each method relative to the best-performing model on each dataset 7
Supplementary Figure S3. Comparison of CrisprPr and other methods on seven test datasets in terms of F1 8
Supplementary Figure S4. Comparison of CrisprPr and other methods on seven test datasets in terms of MCC 9
Supplementary Figure S5. Comparison of CrisprPr and other methods on seven test datasets in terms of AUROC 10
Supplementary Figure S6. Comparison of CrisprPr and its ablated variants on seven test datasets in terms of AUROC 11
Supplementary Figure S7. Performance comparison of M-model, D-model, and their module-removed variants on seven test datasets in terms of AUROC 12
Supplementary Figure S8. Heatmap of mismatch base-pair similarity in the non-seed region of the original MTP embedding space 13
Supplementary Figure S9. Heatmaps of mismatch base-pair similarity in the seed region of the original and updated MTP embedding space 14
Supplementary Figure S10. Heatmap of mismatch base-pair similarity in the non-seed region of the original DRICS embedding space 15

Supplementary Figure S11. Average similarity rankings of update patterns across different datasets in the MTP (a) and DRICS (b) embedding spaces 16
Supplementary Figure S12. Distribution of dataset-level mean adjacent mismatch distances and their association with the contribution of the Inception module 17
Supplementary Figure S13. Performance comparison of CrisprPr under different prior initialization strategies on seven test datasets in terms of AUPRC. 18
Supplementary Table S1. Pairwise counts of shared sgRNAs (≥90% sequence similarity) across datasets. 19
Supplementary Table S2. Precision (P) and recall (R) of off-target prediction methods across seven test datasets. 20
Supplementary Table S3. Comparison of Variances of AUPRC Scores Between CrisprPr and Other Methods. 21
Supplementary Table S4. Similarity of Update Patterns Across Different Datasets in the MTP. 22
Supplementary Table S5. Similarity of Update Patterns Across Different Datasets in the DRICS. 23
References 24

Supplementary Methods

Overall Model Architecture and Processing Pipeline

Both the MTP-based and DRICS-based off-target prediction sub-models adopt the same overall architectural design and processing pipeline. The model takes paired sgRNA–DNA sequences as input and first maps them into prior-informed embedding representations. The embedded features are then fed into an Inception module, which consists of parallel one-dimensional convolutional branches with kernel sizes of 3 and 5 to capture local interaction patterns at different receptive field scales. The outputs of the convolutional branches are concatenated and passed to a BiLSTM module to model long-range dependencies along the sequence. The BiLSTM outputs are subsequently flattened and fed into fully connected layers, which generate the final prediction.

Inception Module

The Inception-based convolutional module takes the prior-informed embedding representations as input and processes them through parallel one-dimensional convolutional branches. Each branch contains a single convolutional layer followed by a ReLU activation[1]. Specifically, one-dimensional convolutions with kernel sizes of 3 and 5 are employed to capture local patterns at different spatial extents along the sequence. All convolutions use a stride of 1 and same padding to preserve the original sequence length. The outputs of the convolutional branches are concatenated along the channel dimension. To retain the original prior information, the concatenated convolutional features are further concatenated with the original embedding representations along the channel dimension. Formally, given the prior-informed embedding representation X, the output of the Inception-based convolutional module can be written as

$$\mathrm{H} = \mathrm{Concat}(\sigma(\mathrm{Conv}_3(\mathrm{X})), \sigma(\mathrm{Conv}_5(\mathrm{X})), \mathrm{X})$$

where $\mathrm{Conv}_\mathrm{k}()$ denotes a one-dimensional convolution with kernel size k, $\sigma()$ denotes the ReLU activation applied after convolution, and $\mathrm{Concat}()$ represents concatenation along the channel dimension. The resulting feature representation H is then passed to the subsequent BiLSTM module.

BiLSTM Module and Output Aggregation

The BiLSTM module takes as input the feature representations produced by the Inception-based convolutional module and is employed to model long-range dependencies along the sgRNA–DNA sequence. We implement the BiLSTM component as a stack of bidirectional LSTM layers applied sequentially. Due to bidirectionality, the feature dimension is doubled after each layer; therefore, the input size of the subsequent layer is updated to twice the hidden size. For the MTP-based sub-model, two stacked BiLSTM layers are used, whereas the DRICS-based sub-model adopts a single BiLSTM layer. Apart from this difference in depth, the BiLSTM modules in both sub-models operate in an identical manner. Given the output of the Inception-based convolutional module H, the stacked BiLSTM then computes

$$\mathrm{Z} = \mathrm{BiLSTM}(\mathrm{H}),$$

where Z represents the sequence of hidden representations obtained by concatenating the forward and backward hidden states at all sequence positions. Finally, the hidden representations at all sequence positions are flattened into a single feature vector:

$$z = \mathrm{Flatten}(Z),$$

which is subsequently fed into fully connected layers to generate the final prediction.

Prediction Head

The flattened feature vector produced by the BiLSTM module is subsequently fed into a fully connected prediction head to generate the final output. The prediction head consists of three fully connected layers with decreasing dimensionality. The first fully connected layer projects flattened feature vector to an 80-dimensional hidden representation, followed by a ReLU activation. The second fully connected layer further reduces the representation to 20 dimensions and is also followed by a ReLU activation. A dropout layer with a dropout rate of 0.35 is then applied to mitigate overfitting. Finally, a third fully connected layer maps the features to a single scalar output.

$$y = \mathrm{Fc}_3(\mathrm{Dropout}(\mathrm{ReLU}(\mathrm{Fc}_2(\mathrm{ReLU}(\mathrm{Fc}_1(Z)))))),$$

where y represents the predicted off-target probability for the given sgRNA-DNA sequence pair. During inference, the average predicted probability of the MTP- and DRICS-based models is converted into a binary classification outcome using a decision threshold. Previous studies have suggested that adjusting the decision threshold is beneficial in class-imbalanced classification tasks[2]. The decision threshold for CrisprPr is selected based on validation data by evaluating candidate thresholds and choosing the one that yields the best validation performance.

Loss Function and Optimization Details

The model is trained using a binary classification objective with binary cross-entropy loss with logits (BCEWithLogitsLoss). Optimization is performed using the Adam optimizer. Training is conducted for a maximum of 300 epochs with a batch size of 50,000. To mitigate overfitting, an early stopping strategy based on validation AUPRC is employed, and the model parameters achieving the best validation performance are retained for subsequent evaluation.

Model Architecture Optimization

The optimization of the model architecture was performed separately for the MTP-based and DRICS-based off-target prediction sub-models. For each sub-model, the optimization procedure was conducted in a two-stage manner:

(1) In the first stage, the structural configurations of the Inception module and the BiLSTM module were optimized. For Inception structure optimization, the BiLSTM depth was fixed to a single layer, and branch combinations were evaluated from four candidate one-dimensional convolutional kernel sizes {1, 2, 3, 5}. The combination yielding the best validation AUPRC was selected as the optimal Inception configuration for the corresponding sub-model.After fixing the Inception branch configuration, the depth of the BiLSTM module was further explored by comparing models with one, two, and three stacked BiLSTM layers. The optimal number of BiLSTM layers was determined based on validation AUPRC and subsequently fixed for each sub-model.

(2) In the second stage, hyperparameters within each module were optimized while keeping the overall architecture fixed. For the Inception module, the number of convolutional filters was selected from {10, 15, 20}. For the BiLSTM module, the hidden size was chosen from {15,

20, 25}. All combinations of these hyperparameters were evaluated independently for each sub-model, and the optimal configuration was selected according to validation AUPRC.

Throughout the entire optimization process, model selection was consistently based on validation AUPRC to ensure fair comparison and robustness under class-imbalanced conditions.

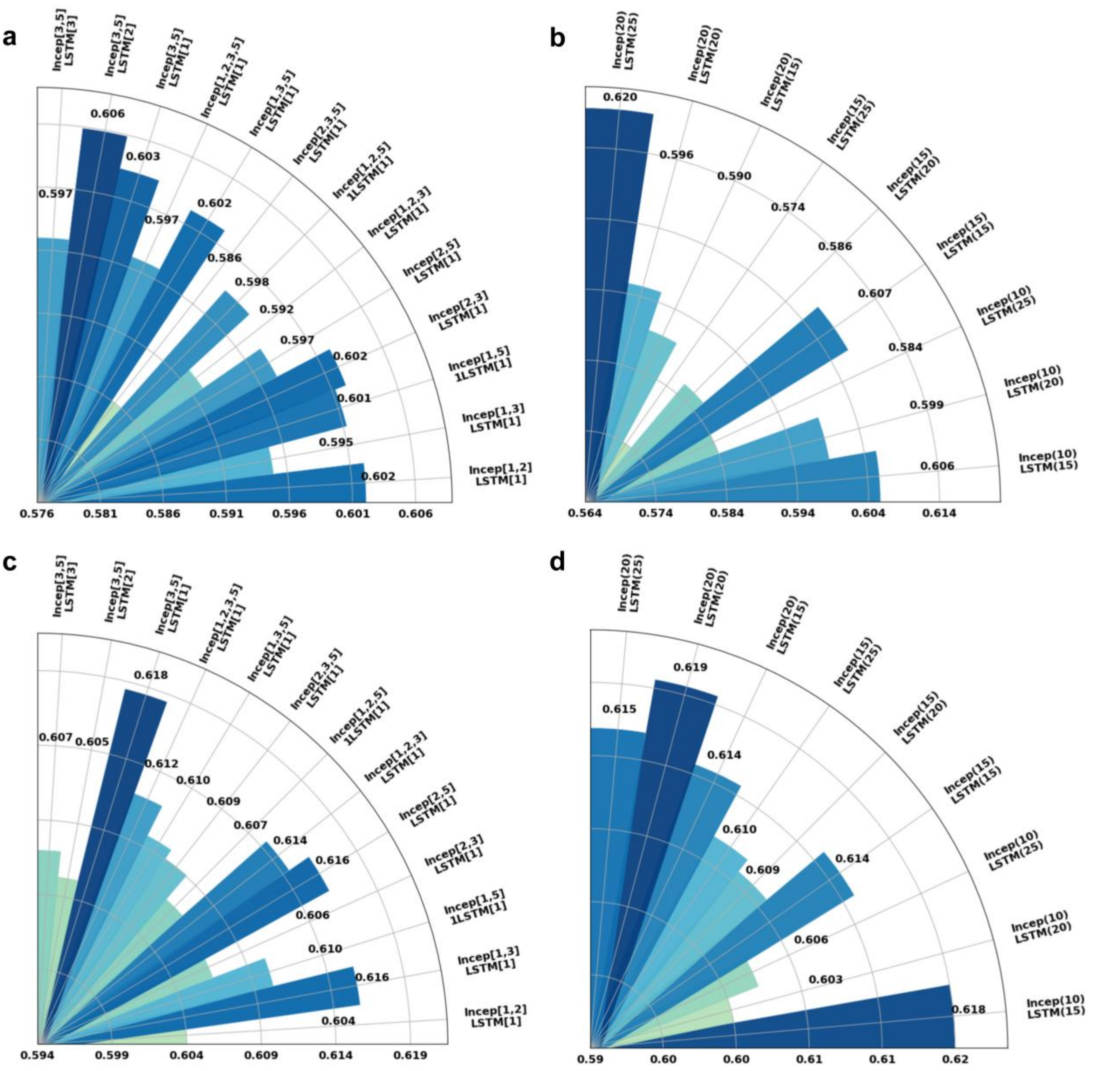

Supplementary Figure S1. Hyperparameter optimization of the M-model (a,b) and D-model (c,d) architectures. Models were trained on the training set, and hyperparameters were selected according to the validation AUPRC results on the TTISS-CT dataset. In (a) and (c), Incep[·] denotes the set of kernel sizes used by the multi-branch Inception module (e.g., Incep[3,5] uses kernel sizes 3 and 5); Lstm[·] denotes the number of stacked BiLSTM layers (e.g., Lstm[2]). In (b) and (d), Incep(·) denotes the number of convolution filters per branch (e.g., Incep(10) = 10 filters per branch); Lstm(·) denotes the number of hidden units per LSTM layer (e.g., Lstm(25)). When optimizing kernel-size combinations and BiLSTM depth, the number of convolution filters and LSTM units were fixed to the default CRISPR-net settings (10 filters and 15 units, respectively). Conversely, when tuning filter counts and hidden units, kernel-size combinations and BiLSTM layers were fixed to the optimal configuration from the previous validation stage.

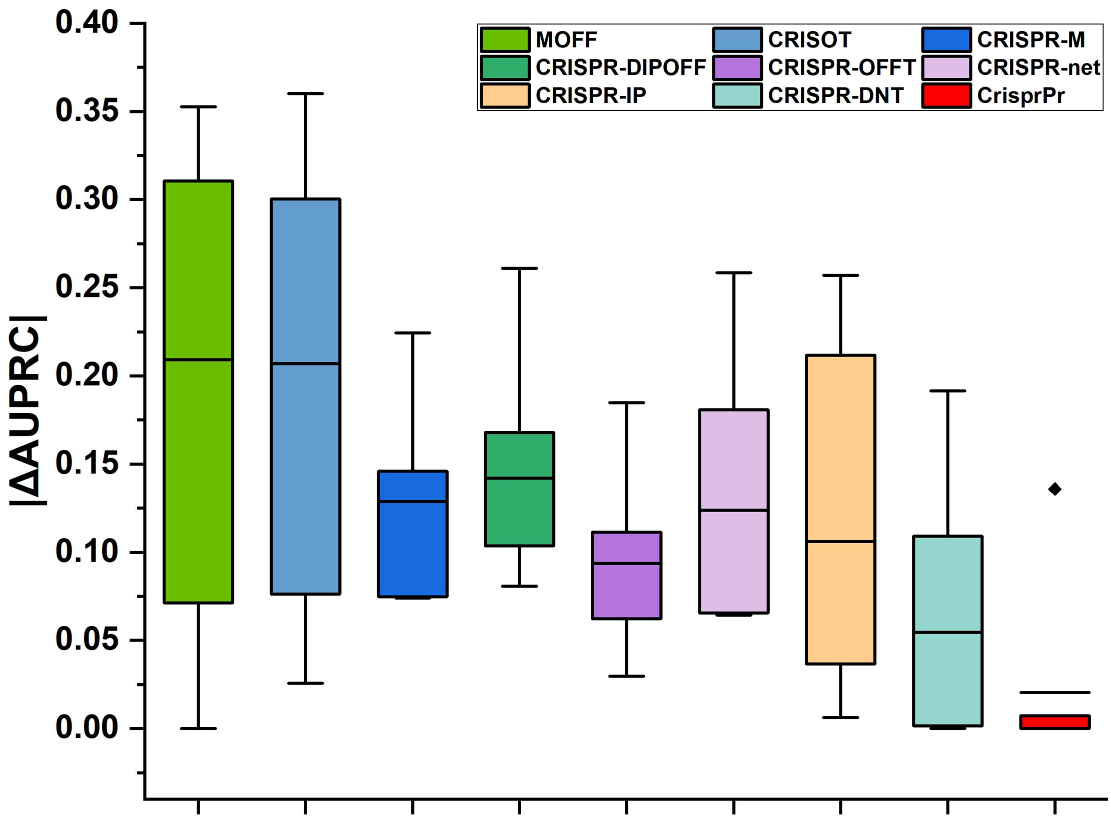

Supplementary Figure S2. Performance gap of each method relative to the best-performing model on each dataset. For each method, the absolute AUPRC difference from the best-performing model on each dataset was computed across the seven evaluation datasets. The central line represents the mean value, while the bottom and top of the vertical line denote the minimum and maximum values, respectively.

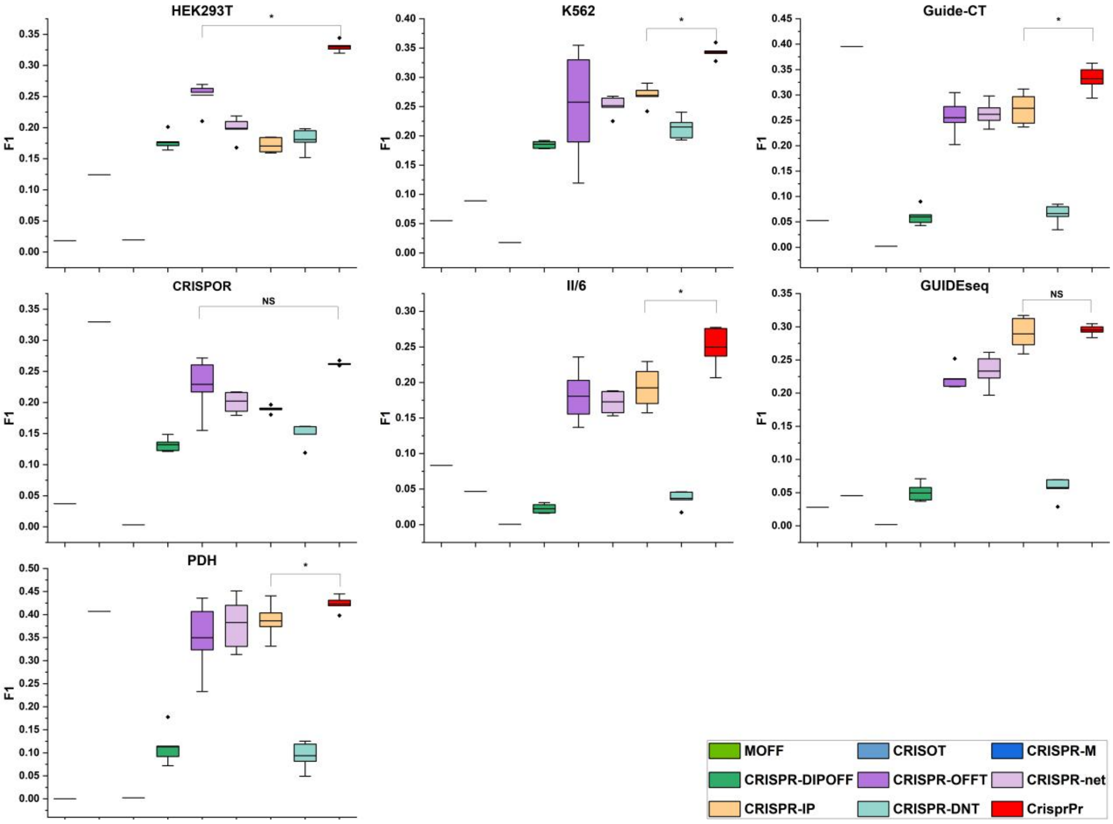

Supplementary Figure S3. Comparison of CrisprPr and other methods on seven test datasets in terms of F1. The compared methods are ordered as follows: MOFF, CRISOT, CRISPR-M, CRISPR-DIPOFF, CRISPR-OFFT, CRISPR-net, CRISPR-IP, CRISPR-DNT, and CrisprPr. The central line represents the mean value, while the bottom and top of the vertical line denote the minimum and maximum values, respectively. Significance: *, $p < 0.05$; NS, $p \geq 0.05$ (Wilcoxon signed-rank test).

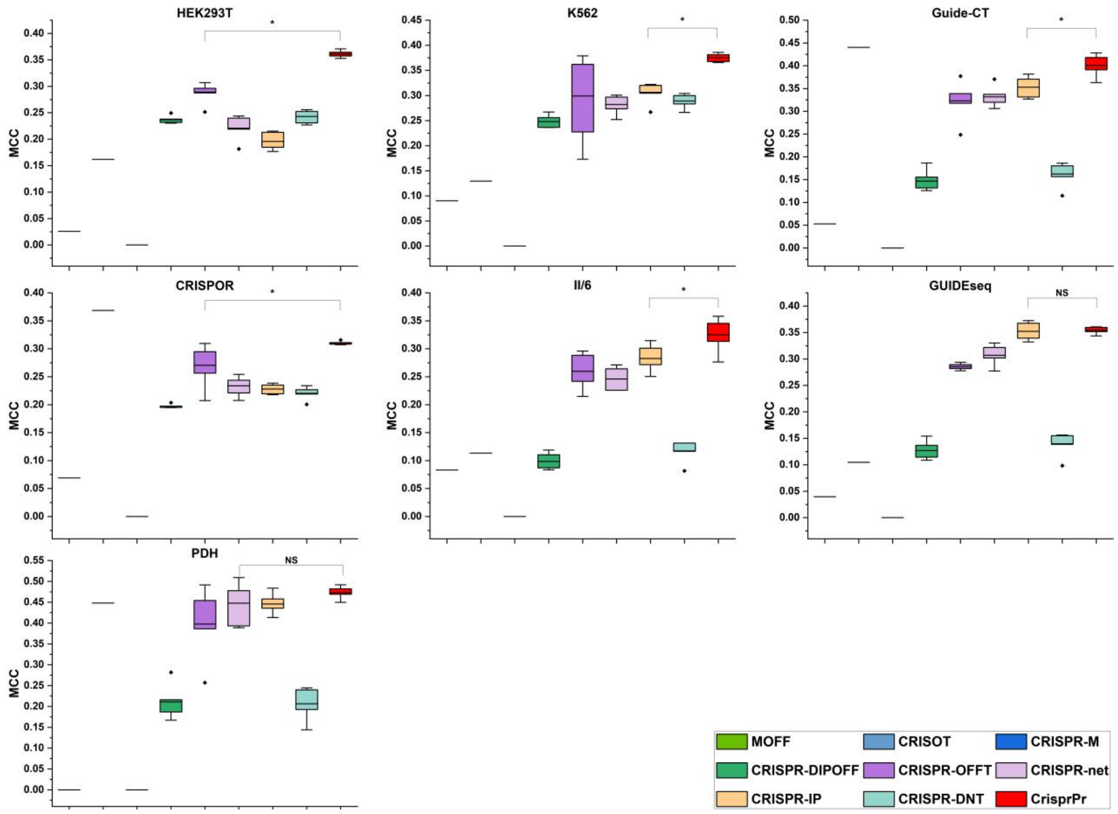

Supplementary Figure S4. Comparison of CrisprPr and other methods on seven test datasets in terms of MCC. The compared methods are ordered as follows: MOFF, CRISOT, CRISPR-M, CRISPR-DIPOFF, CRISPR-OFFT, CRISPR-net, CRISPR-IP, CRISPR-DNT, and CrisprPr. The central line represents the mean value, while the bottom and top of the vertical line denote the minimum and maximum values, respectively. Significance: *, $p < 0.05$; NS, $p \geq 0.05$ (Wilcoxon signed-rank test).

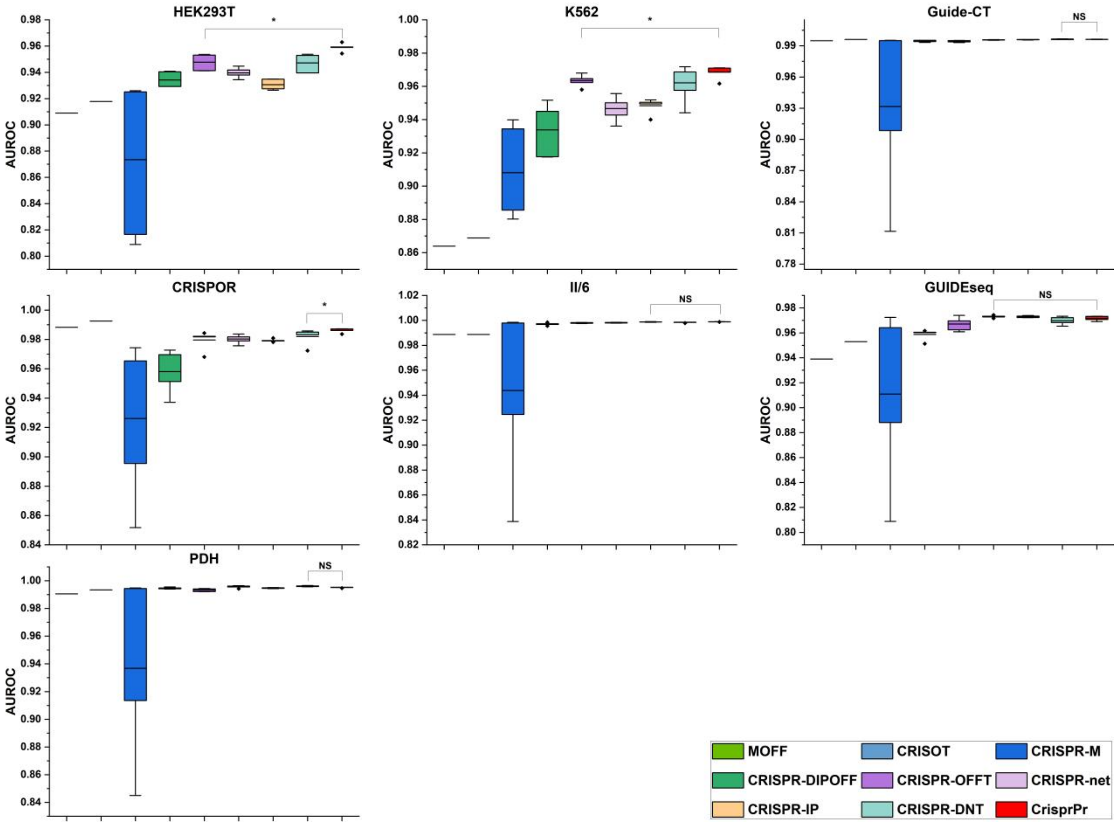

Supplementary Figure S5. Comparison of CrisprPr and other methods on seven test datasets in terms of AUROC. The compared methods are ordered as follows: MOFF, CRISOT, CRISPR-M, CRISPR-DIPOFF, CRISPR-OFFT, CRISPR-net, CRISPR-IP, CRISPR-DNT, and CrisprPr. The central line represents the mean value, while the bottom and top of the vertical line denote the minimum and maximum values, respectively. Significance: *, $p < 0.05$; NS, $p \geq 0.05$ (Wilcoxon signed-rank test).

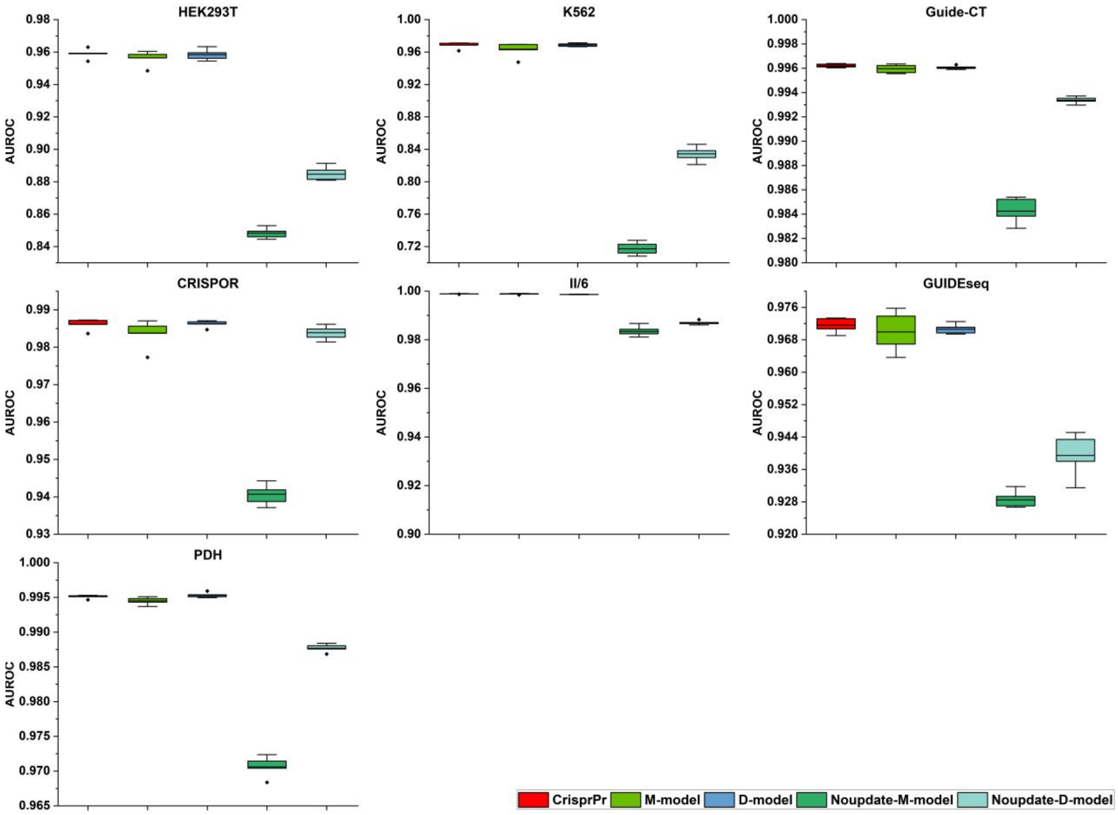

Supplementary Figure S6. Comparison of CrisprPr and its ablated variants on seven test datasets in terms of AUROC. The compared methods are ordered as follows: CrisprPr, M-model, D-model, Noupdate-M-model and Noupdate-D-model. The central line represents the mean value, while the bottom and top of the vertical line denote the minimum and maximum values, respectively.

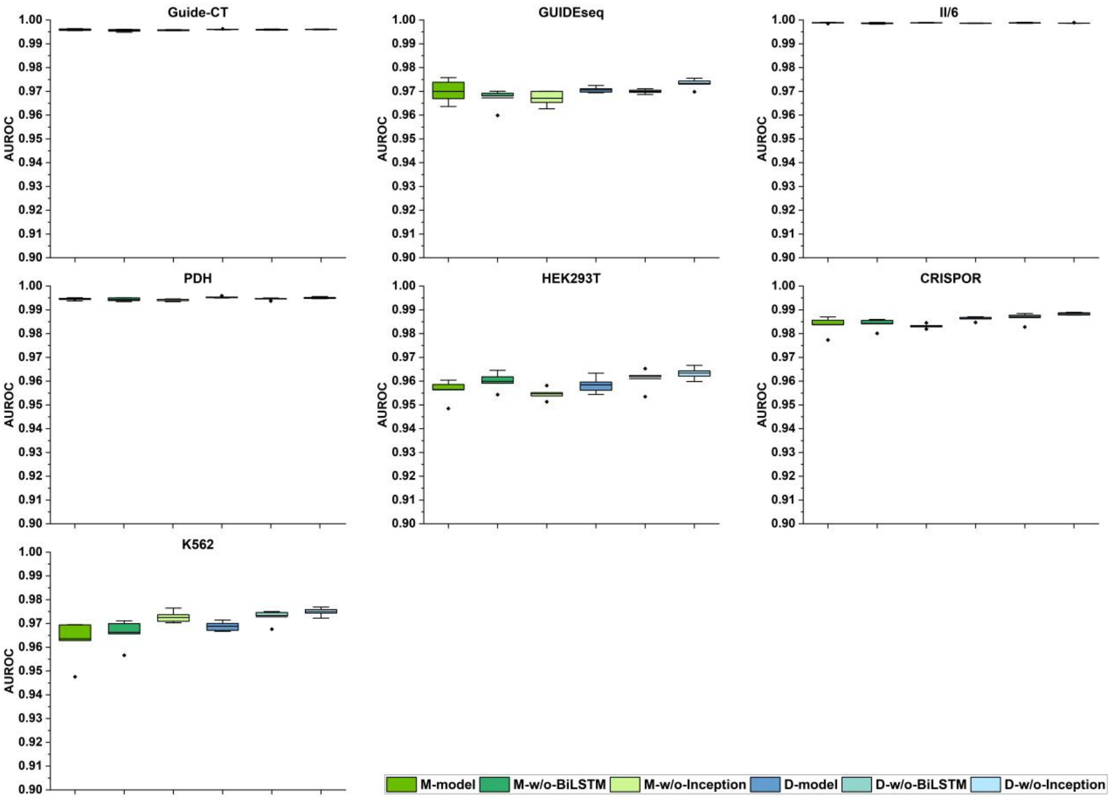

Supplementary Figure S7. Performance comparison of M-model, D-model, and their module-removed variants on seven test datasets in terms of AUROC. The compared methods are ordered as follows: M-model, M-w/o-BiLSTM, M-w/o-Inception, D-model, D-w/o-BiLSTM and D-w/o-Inception. The central line represents the mean value, while the bottom and top of the vertical line denote the minimum and maximum values, respectively.

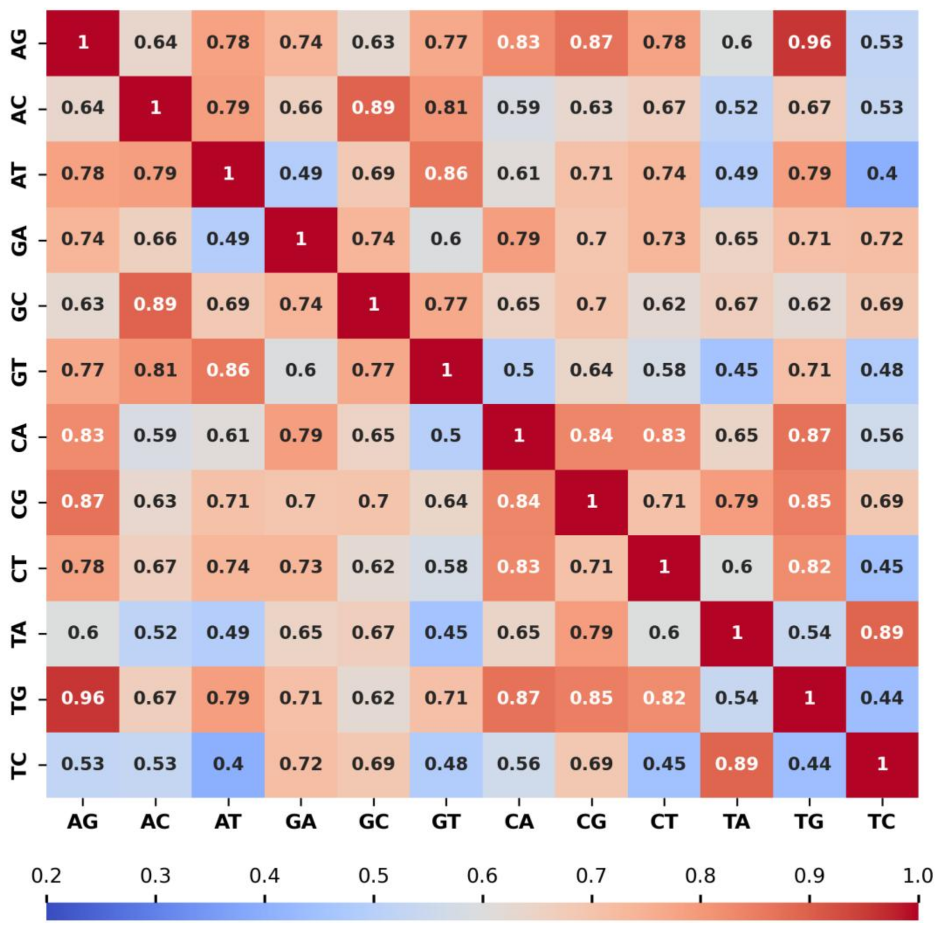

Supplementary Figure S8. Heatmap of mismatch base-pair similarity in the non-seed region of the original MTP embedding space. Similarity was assessed using a weighted combination of Euclidean distance and Pearson correlation coefficient.

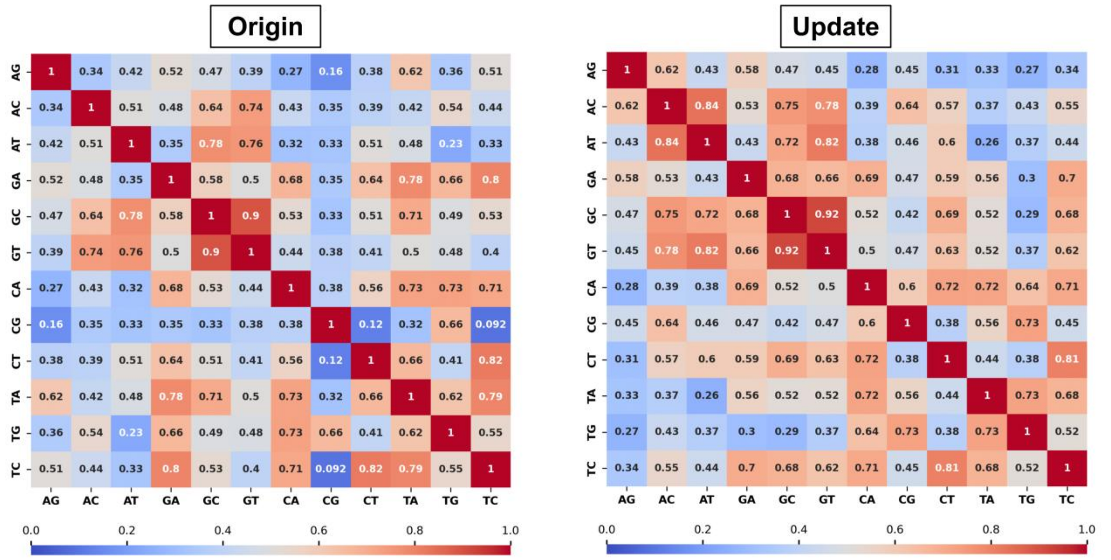

Supplementary Figure S9. Heatmaps of mismatch base-pair similarity in the seed region of the original and updated MTP embedding space. Similarity was assessed using a weighted combination of Euclidean distance and Pearson correlation coefficient.

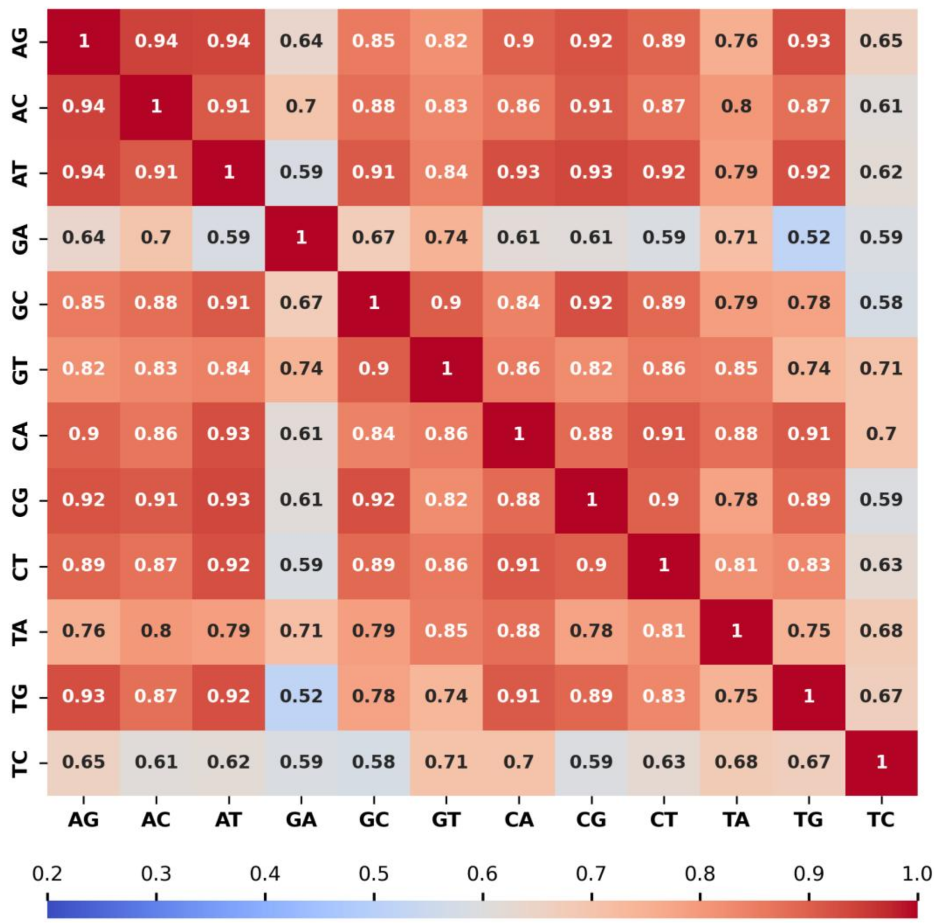

Supplementary Figure S10. Heatmap of mismatch base-pair similarity in the non-seed region of the original DRICS embedding space. Similarity was assessed using a weighted combination of Euclidean distance and Pearson correlation coefficient.

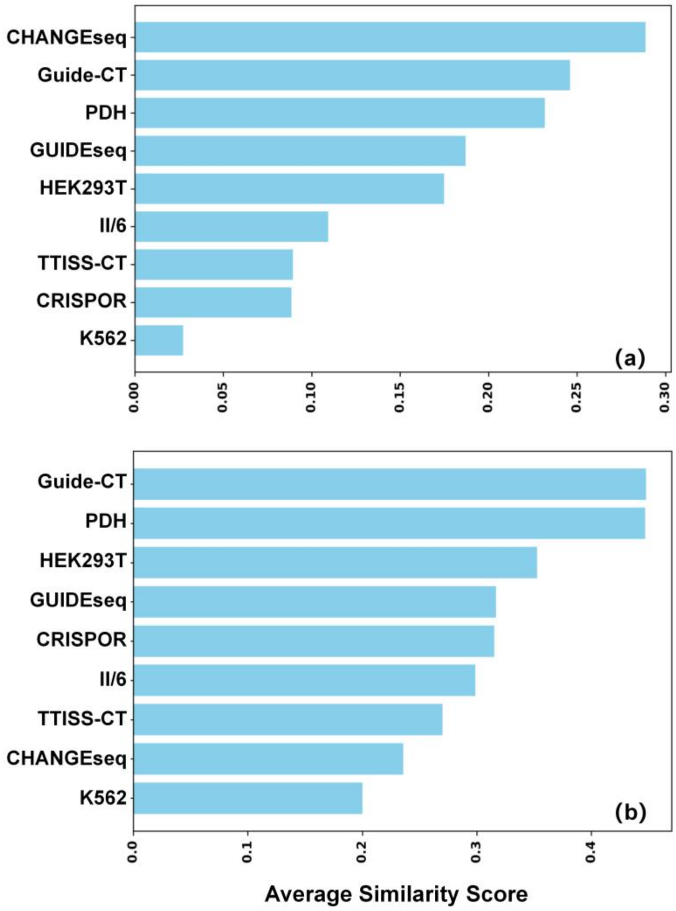

Supplementary Figure S11. Average similarity rankings of update patterns across different datasets in the MTP (a) and DRICS (b) embedding spaces. For each dataset, the average similarity was computed as the mean pairwise Pearson correlation between its fused embedding-space variation and those of all other datasets.

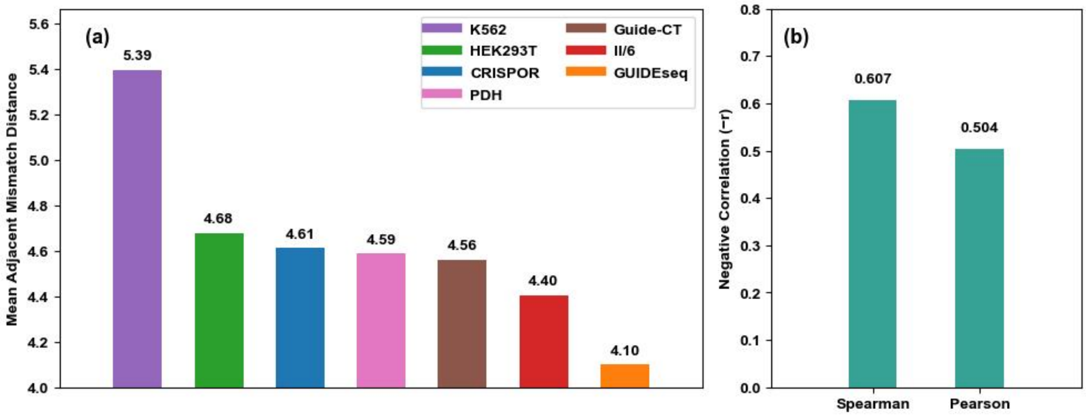

Supplementary Figure S12. Distribution of dataset-level mean adjacent mismatch distances and their association with the contribution of the Inception module. (a) Mean base-pair distance between adjacent mismatches within true off-target (positive) sgRNA-target pairs across seven test datasets. (b) Negative correlation (−r) between the mean mismatch distance and the performance gain contributed by the Inception module ($\Delta\_\mathrm{Inception}$), measured using Spearman and Pearson correlation coefficients. $\Delta\_\mathrm{Inception}$ is defined as $\Delta\_\mathrm{Inception} = \mathrm{AUPRC}_{\mathrm{M-model}} - \mathrm{AUPRC}_{\mathrm{M-w/o-Inception}}$.

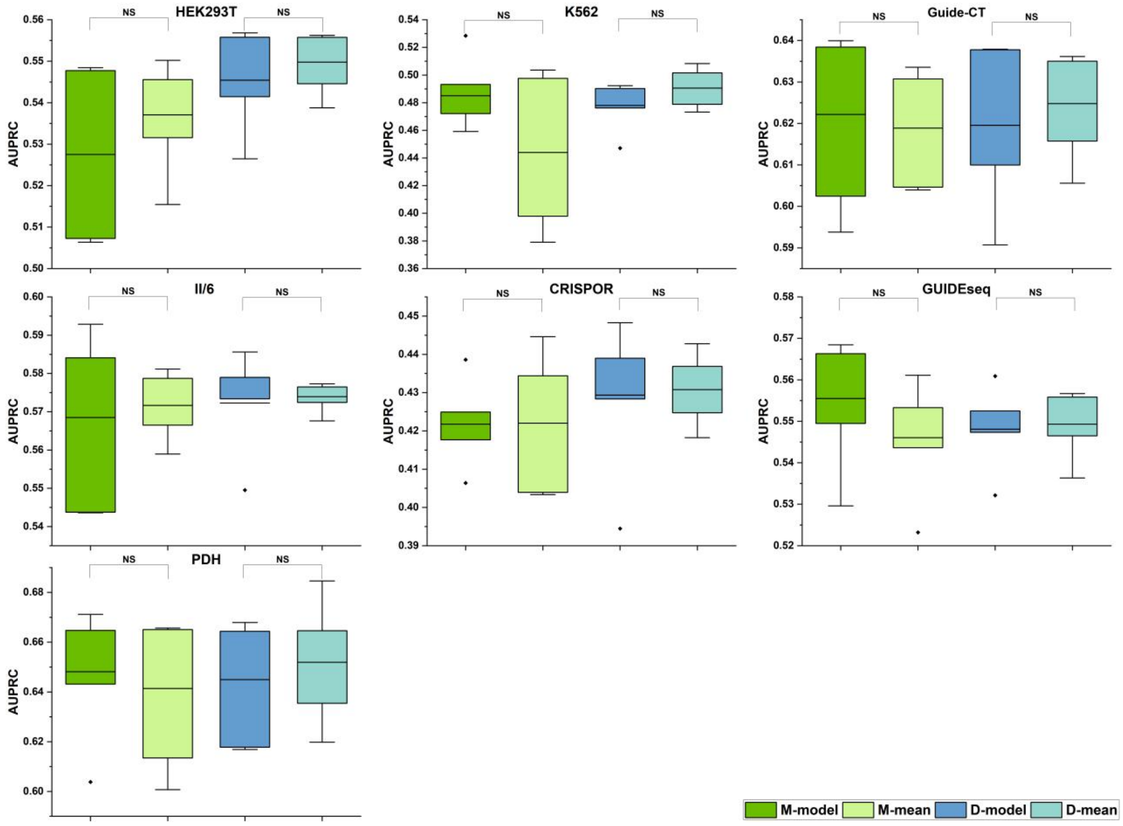

Supplementary Figure S13. Performance comparison of CrisprPr under different prior initialization strategies on seven test datasets in terms of AUPRC. D-model and M-model denote variants using binary initialization (missing entries filled with 0 or 1), while D-mean and M-mean use mean-value initialization (missing entries filled with the global mean value)**.** The central line represents the mean value, while the bottom and top of the vertical line denote the minimum and maximum values, respectively. Significance: *, $p < 0.05$; NS, $p \geq 0.05$ (Wilcoxon signed-rank test).

Supplementary Table S1. Pairwise counts of shared sgRNAs (≥90% sequence similarity) across datasets.

	CHANGEseq	TTISS-CT	GUIDEseq	HEK293T	II/6	K562	CRISPOR	Guide-CT	PDH
**CHANGEseq**	-	0	57	0	0	0	0	0	0
**TTISS-CT**	0	-	0	0	0	0	0	0	0
**GUIDEseq**	57	0	-	0	0	0	0	0	0
**HEK293T**	0	0	0	-	0	1	16	9	9
**II/6**	0	0	0	0	-	0	0	22	0
**K562**	0	0	0	1	0	-	12	1	1
**CRISPOR**	0	0	0	16	0	12	-	10	9
**Guide-CT**	0	0	0	9	22	1	10	-	9
**PDH**	0	0	0	9	0	1	9	9	-

Supplementary Table S2. Precision (P) and recall (R) of off-target prediction methods across seven test datasets.

		GUIDE seq	HEK293T	II/6	K562	CRISP OR	Guide-CT	PDH
**MOFF**	**P**	0.1114	0.1152	0.0833	0.2727	0.2692	0.0526	0.0000
	**R**	0.0206	0.0182	0.0135	0.0833	0.0310	0.0526	0.0000
**CRISOT**	**P**	0.0283	0.0836	0.0249	0.0512	0.3840	0.3781	0.3633
	**R**	0.4554	0.6962	0.5408	0.6357	0.4653	0.6268	0.6490
**CRISPR-M**	**P**	0.0010	0.0100	0.0003	0.0090	0.0016	0.0011	0.0012
	**R**	1.0000	1.0000	1.0000	1.0000	1.0000	1.0000	1.0000
**CRISPR-DIPOFF**	**P**	0.0270	0.1249	0.0114	0.1372	0.0941	0.0341	0.0666
	**R**	0.6594	0.8812	0.6788	0.9872	0.6767	0.9823	0.9721
**CRISPR-OFFT**	**P**	0.1771	0.2949	0.1324	0.3444	0.3284	0.2161	0.3305
	**R**	0.3904	0.6486	0.4514	0.7119	0.4068	0.7003	0.6645
**CRISPR-net**	**P**	0.1775	0.2333	0.1173	0.3861	0.2942	0.1925	0.2974
	**R**	0.2804	0.7352	0.2989	0.6692	0.2862	0.7190	0.7988
**CRISPR-IP**	**P**	0.2359	0.2363	0.1337	0.4923	0.3673	0.1969	0.2869
	**R**	0.2297	0.6873	0.2524	0.7901	0.2358	0.7971	0.8011
**CRISPR-DNT**	**P**	0.0314	0.1167	0.0194	0.1689	0.1116	0.0357	0.0502
	**R**	0.6996	0.8999	0.7216	0.9671	0.7372	0.9743	0.9892
**CrisprPr**	**P**	0.2434	0.3794	0.1950	0.3214	0.2940	0.2988	0.3897
	**R**	0.4663	0.6739	0.4463	0.7279	0.5349	0.7271	0.7149

Supplementary Table S3. Comparison of Variances of AUPRC Scores Between CrisprPr and Other Methods.

	CRISPR_M	CRISPR-DIPOFF	CRISPR-OFFT	CRISPR-net	CRISPR-IP	CRISPR-DNT	CrisprPr
**HEK293T**	0.005904	0.000713	0.000305	0.000316	0.000081	0.000882	0.000106
**K562**	0.004953	0.001915	0.000831	0.000603	0.000270	0.001306	0.000170
**Guide-CT**	0.002752	0.000070	0.000485	0.001810	0.000162	0.000186	0.000029
**CRISPOR**	0.003229	0.000396	0.000331	0.000293	0.000056	0.000867	0.000061
**II/6**	0.001617	0.000315	0.000467	0.001637	0.000216	0.000268	0.000064
**GUIDEseq**	0.001387	0.000141	0.000420	0.000401	0.000091	0.000049	0.000031
**PDH**	0.005399	0.000534	0.003725	0.003157	0.000607	0.000891	0.000061

Supplementary Table S4. Similarity of Update Patterns Across Different Datasets in the MTP.

	CHANGEseq	Guide-CT	PDH	HEK293T	GUIDEseq	II/6	TTISS-CT	K562	CRISPOR
**CHANGEseq**	-	0.1939	0.2235	0.3469	0.5449	0.1723	0.3278	0.1494	0.3511
**Guide-CT**	0.1939	-	0.6715	0.3422	0.1113	0.4718	-0.0057	0.0888	0.0933
**PDH**	0.2235	0.6715	-	0.4449	0.0675	0.3404	-0.0388	0.1663	-0.0212
**HEK293T**	0.3469	0.3422	0.4449	-	0.1385	0.0861	-0.0096	0.0439	0.0053
**GUIDEseq**	0.5449	0.1113	0.0675	0.1385	-	0.0666	0.2865	-0.0652	0.3462
**II/6**	0.1723	0.4718	0.3404	0.0861	0.0666	-	-0.06	-0.0048	-0.1981
**TTISS-CT**	0.3278	-0.0057	-0.0388	-0.0096	0.2865	-0.06	-	-0.0389	0.2535
**K562**	0.1494	0.0888	0.1663	0.0439	-0.0652	-0.0048	-0.0389	-	-0.1213
**CRISPOR**	0.3511	0.0933	-0.0212	0.0053	0.3462	-0.1981	0.2535	-0.1213	-

Supplementary Table S5. Similarity of Update Patterns Across Different Datasets in the DRICS.

	CHANGEseq	Guide-CT	PDH	HEK293T	GUIDEseq	II/6	TTISS-CT	K562	CRISPOR
**CHANGEseq**	-	0.2598	0.2554	0.1547	0.4613	0.1772	0.2995	0.0837	0.1952
**Guide-CT**	0.2598	-	0.8555	0.5402	0.3541	0.4784	0.3101	0.2586	0.5249
**PDH**	0.2554	0.8555	-	0.5322	0.4126	0.4068	0.3613	0.2792	0.4734
**HEK293T**	0.1547	0.5402	0.5322	-	0.3206	0.3091	0.2855	0.196	0.4832
**GUIDEseq**	0.4613	0.3541	0.4126	0.3206	-	0.2215	0.382	0.1057	0.276
**II/6**	0.1772	0.4784	0.4068	0.3091	0.2215	-	0.2117	0.4141	0.1698
**TTISS-CT**	0.2995	0.3101	0.3613	0.2855	0.382	0.2117	-	0.0882	0.2226
**K562**	0.0837	0.2586	0.2792	0.196	0.1057	0.4141	0.0882	-	0.1758
**CRISPOR**	0.1952	0.5249	0.4734	0.4832	0.276	0.1698	0.2226	0.1758	-

References

[1] Lin J, Zhang Z, Zhang S, et al. CRISPR‐net: a recurrent convolutional network quantifies CRISPR off‐target activities with mismatches and indels[J]. Advanced science, 2020, 7(13): 1903562.

[2] Yang Y, Zhang R, Singh S, et al. Exploiting sequence-based features for predicting enhancer–promoter interactions[J]. Bioinformatics, 2017, 33(14): i252-i260.
